# Hepatocellular Carcinoma associated with Extra-hepatic Primary Malignancy: its Secular change, Clinical Manifestations and Survival

**DOI:** 10.1038/srep30156

**Published:** 2016-07-22

**Authors:** Kwong Ming Kee, Jing-Houng Wang, Chih-Chi Wang, Yu-Fan Cheng, Sheng-Nan Lu

**Affiliations:** 1 Division of Hepatogastroenterology, Department of Internal Medicine, Kaohsiung Chang Gung Memorial Hospital and Chang Gung University College of Medicine, Kaohsiung, Taiwan; 2 Department of Surgery, Kaohsiung Chang Gung Memorial Hospital and Chang Gung University College of Medicine, Kaohsiung, Taiwan; 3 Department of Radiology, Kaohsiung Chang Gung Memorial Hospital and Chang Gung University College of Medicine, Kaohsiung, Taiwan

## Abstract

Clinical manifestations between hepatocellular carcinoma (HCC) and extra-hepatic primary malignancy (EHPM) are lack of large-scale study. We enrolled 14555 HCC patients between 1986 and 2013 retrospectively. The EHPM was classified as prior, synchronous and metachronous group based on before, within and after 6 months of HCC diagnosis, respectively. The incidence rate of EHPM is 3.91% (95% confidence interval [CI]: 3.60–4.23%). Urogenital cancers, kidney and bladder, were at unexpected higher ranks. Older in age, Child-Pugh A cirrhosis, negativity of HBsAg and anti-HCV, and earlier BCLC staging are independent factors associated with EHPM. The survival rates of EHPM improve over time and also better than HCC-alone. Cox proportional-hazards regression shows independent poor prognostic factors are age >60, male, AFP levels ≥400 ng/ml, positivity of HBsAg, Child-Pugh B vs. A, Non-metachronous group, respectively, treated with local ablation, transcatheter arterial embolization, radiotherapy and supportive care vs. surgery, respectively, TNM stage IIIA vs. I, and BCLC stages A, B, C and D vs. 0, respectively. Survival of EHPM improve could be explained by early diagnosis and improve treatment of cancers.

Due to the early diagnosis and improvement of treatment of cancers, the overall survival rates in cancer survivors are increasing. In addition, the incidence of second cancers was also increases in the USA^1^. Hepatocellular carcinoma (HCC) ranks as the sixth most common cancer worldwide and the third deadliest form of cancer overall^2^, was also one of the most common causes of cancer in Taiwan. Early detection of small HCC, improve HCC treatment and antiviral therapy for chronic hepatitis B and C in Taiwan^3^ had been improved the survival rates of HCC in recent decades.

Some previous studies had investigated in the incidence, clinical manifestation and survival of extra-hepatic primary malignancy (EHPM) associated with HCC^4^,^5^,^6^,^7^,^8^,^9^,^10^,^11^,^12^,^13^,^14^,^15^,^16^,^17^,^18^,^19^. Majority of the studies only included small amount of cases. The EHPM case number was ranging from seven to seventy-four cases, whereas EHPM prevalence rates were 2.1~25.7%. Large-scale study in incidence, survival and clinical manifestations of EHPM in HCC survivors were rarely reported. These might be partly related to the high mortality rate due to advanced stage when HCC diagnosis and poor response to treatment in past two decades.

Several reports showed that mean age of EHPM group were older^8^,^10^,^20^, whereas some studies showed no significant difference in age^9^,^14^,^16^,^17^. Some studies showed no significant difference in gender^9^,^13^,^14^,^16^,^17^. In liver related variables, some studies showed no difference in prevalence of cirrhosis^9^,^13^,^14^,^17^, alanine aminotransferase (ALT)^13^,^14^ or prothrombin time^13^,^14^, whereas some studies showed lower rates of cirrhosis^8^,^11^ and lower aspartate transaminase (AST) level^14^ in EHPM group. No difference of alpha-fetoprotein (AFP) level in some studies between EHPM and HCC-alone groups^9^,^13^,^14^. In most prior studies, survival rates between EHPM and HCC-alone groups showed no significantly difference^8^,^10^,^12^,^13^,^16^, whereas a study that enrolled patients who receiving curative hepatectomy had better survival in EHPM group^17^. However, the majority of the studies showed no significant difference in clinical variables and survival rates.

Many heterogeneity factors existed in previous EHPM studies, such as difference in diagnostic time, different study designs and vary common cancer sites of EHPM. The associated factors and survival rates of EHPM were also difficult to make conclusion from these studies due to relative small scale of sample size. Until now, there was no large hospital-based study to investigate the incidence, risk factors, prognosis and survival of EHPM. A recent nationwide study in Taiwan had enrolled a large scale population into the study to investigate the risk of second primary cancers associated with HCC^20^. However, the data which obtained from the National Health Insurance Research database of Taiwan was lack of clinical manifestation, laboratory data, image and pathological information. In addition, the associated factors, secular change and survival rates of EHPM were not further analyzed. The aim of the study was to analyze the incidence, clinical manifestations, prognosis and survival of EHPM based on HCC patients in our hospital.

## Patients and Methods

Between 1986 and 2013, a total of 14555 patients diagnosed as HCC in Kaohsiung Chang Gung Memorial hospital were enrolled into the study.

### Diagnosis of HCC

We setup HCC database since 2003, the diagnostic criteria between 1986 and 2004 was based on the guidelines of European Association of the Study of the Liver (EASL) that published in 2001^21^. The diagnostic criteria of HCC were arbitrarily classified as criterion 1 indicated diagnosis of HCC verified by either pathology or cytology (n = 3506, 24.1%). Criterion 2 was AFP level >400 ng/ml plus at least one image study showing a typical HCC image (n = 2599, 17.9%). Criterion 3 was initially did not fit criteria 1 or 2, but did fit either criteria 1 or 2 during the follow-up period (n = 269, 1.8%). Criterion 4 was based on typical image studies but did not fit criteria 1 to 3 (n = 2298, 15.8%). The diagnostic criteria were updated based on practice guidelines of American Association for the Study of Liver Disease (AASLD) after 2005^22^. Between 2005 and 2013, criterion 1 was verified by either pathology or cytology (n = 3343, 23.0%). Criterion 2 was cirrhotic background, tumor size 1–2 cm, and two typical dynamic image studies (n = 258, 1.8%). Criterion 3 was cirrhosis, tumor size >2 cm and one typical image (n = 2234, 15.3%). Criterion 4 was cirrhosis, tumor size >2 cm, and one typical image plus AFP level >200 ng/ml (n = 48, 0.3%).

### Diagnosis and classifications of EHPM

All patients were reviewed retrospectively for diagnosis of second primary cancer. The diagnosis of cancer was confirmed by practice guideline. The definition was the tumor has definite diagnosis of malignancy, the tumor must be histological distinct and the possibility of metastasis of the other must be excluded^23^. The EHPM was classified to subgroups as prior, synchronous and metachronous group based on before, within and after 6 months of HCC diagnosis, respectively.

## Methods

HCC staging system for analysis included 7th edition tumor-node-metastasis (TNM)^24^ and Barcelona Clinic Liver Cancer (BCLC) staging systems^25^. Initial treatment modalities included liver transplantation, surgical resection, percutaneous local ablation, transcatheter arterial embolization (TAE), radiotherapy, systemic chemotherapy, supportive care and etc. All patients were followed-up until death, loss follow-up or the end of May 2014. The underlying cause of death was classified according to the death certificate data. The study protocol was approved by the Institutional Review Board of Chang Gung Memorial Hospital, Taiwan.

The incidence rates, clinical characteristic, risk factors and survival rates over time between EHPM and HCC were compared. We further matching sex and age to analyze the clinical manifestation and risk factors of EHPM

### Statistical analysis

Cumulative survival rates were analyzed by the Kaplan–Meier curves, and the differences between survival curves and linear trends in groups and subgroups were statistically compared by log-rank test. We analyzed the survival rates after excluding patients who survived less than 6 months, to avoid the influence of metachronous group that was defined as at least 6 months after HCC diagnosis. The χ2 analysis, Fisher’s exact test, and Student’s t test were used for statistical evaluation, as appropriate. A value of P < 0.05 was considered statistically significant. Statistical analysis was performed using SPSS 17 for Windows (SPSS Inc., Chicago, IL, USA) and SigmaStat^®^ 3.1. Survival curves were constructed using SigmaPlot^®^ 9.0. Secular trends in incidence of EHPM based on annual data were tested with a simple log-linear regression model. The model estimates the average annual per cent change (AAPC) in rates with time periods. A two-tailed test of statistical significance was applied to the AAPC^26^. We analyzed the effects of chronological age, time period on incidence trends in EHPM. Cases were grouped into 5-year age groups. To obtain the effects of age, period and cohort on breast cancer incidence, models were fitted on the assumption that the number of cases constituted a variable with a Poisson distribution.

## Results

A total of 570 cases were diagnosed as EHPM, overall incidence rates are 3.91% (95% confidence interval [CI]:3.60–4.23%). Table 1 shows age-specific incidence rates of EHPM between 1986 and 2013. Table 2 shows age-specific AAPC for 1986–2013. The overall age-standardized incidence increased annually by 4.9% during the study period. The incidence rates increased significantly in age period 45–49, 50–54, 55–59 and 60–64. The cancer sites of EHPM based on diagnostic time were listed in Table 3. Overall, the six most common EHPM cancer sites were colon, kidney, bladder, oropharynx, lung and gastric cancers. There were 240 (1.65%), 149 (1.02%) and 181 (1.24%) cases in prior, synchronous and metachronous group, respectively. By gender, the six most common male cancer sites were oropharynx (n = 61), colon (n = 56), bladder (n = 51), kidney (n = 42), gastric (n = 39) and lung cancers (n = 38). The six most common female cancers site were kidney (n = 32), colon (n = 25), breast (n = 24), cervix (n = 19), bladder (n = 16) and lung cancers (n = 9).

### Factors associated with extra-hepatic primary malignancy

Table 4 shows univariate analysis of clinical manifestation between EHPM and HCC-alone groups. In EHPM group, there were older in age, female predominant, lower rates of elevated AST and ALT levels, AFP level ≥400 ng/ml, and positivity of HBsAg, higher rates of Child-Pugh A, albumin levels ≥3.5 g/dl, bilirubin levels <2 mg/dl, and earlier stage of TNM (stage I) and BCLC staging (stage A) (all p < 0.05). For initial treatment of HCC, EHPM group had higher rates of receiving curative treatment, including surgery and local ablation, and lower rates of supportive treatment (p < 0.001). After matching with age and sex, there were still almost same significant factors between two groups, except initial treatment of HCC (p = 0.083).

Table 5 shows multivariate analysis to identify the independent factors associated with EHPM. The results showed that age > 60 (odds ratio [OR], 1.74; 95% CI, 1.43–2.10), AST level < 40 IU/L (1.32; 1.08–1.61), Child-Pugh A (1.43; 1.14–1.80), AFP < 400 ng/ml (1.38; 1.11–1.72), negativity of anti-HCV (1.49; 1.20–1.87) and HBsAg (1.70; 1.36–2.11), and BCLC stage 0 (2.56; 1.32–4.99), A (3.42; 1.87–6.24), B (2.27; 1.24–4.15) and C (1.73; 0.94–3.16) vs. D, respectively, are independent factors. After matching sex and age, the results showed ALT levels <40 IU/L (1.43; 1.18–1.74), Child-Pugh A (1.46; 1.15–1.87) and BCLC staging are independent factors associated with EHPM.

### Survival rates of extra-hepatic primary malignancy and its associated factors

**Figure 1:**
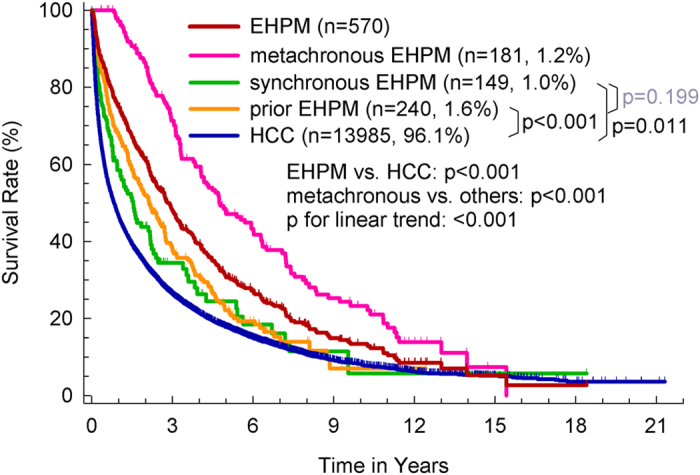
The survival rates of HCC, overall EHPM and EHPM subgroups. The survival rates of EHPM (n = 570) is significantly better than HCC alone (n = 13985) (p < 0.001). The survival rates of metachronous group was higher than prior, synchronous EHPM and HCC-alone groups (all p < 0.001). The survival rates of prior (p < 0.001) and synchronous (p = 0.011) groups were also higher than HCC-alone group.

Figure 1 shows the survival rates of HCC, EHPM and its subgroups. Overall, the survival rate of EHPM group is better than HCC-alone group (p < 0.001). The overall survival rates of EHPM and HCC-alone groups at year-1, -3 and -5 was 75.0%, 48.0%, 30.7% and 46.6%, 26.4%, and 17.8%, respectively (P < 0.001). Median survival time of EHPM and HCC-alone group was 2.8 and 0.83 years, respectively. The underlying cause of death was classified according to the death certificate data. The percentage of HCC/liver and EHPM related death were 85.3% in HCC-alone group, 64.8% and 19.4% in prior group, 48.7% and 29.4% in synchronous group, and 58% and 24.7% in metachronous group, respectively.

The survival rates of metachronous group was better than prior, synchronous and HCC-alone groups (all p < 0.001). The survival rates of prior (p < 0.001) and synchronous (p = 0.011) groups were better than HCC-alone group. The median survival time of metachronous, prior, synchronous and HCC-alone groups, was 4.74, 2.14, 1.54, and 0.83 years, respectively, with a decreasing linear trend (p < 0.001). After excluding patients that survived less than 6 months, the survival rates of metachronous group better than prior, synchronous and HCC-alone groups (all p < 0.001), with a significant decreasing linear trends (p < 0.001).

Table 6 shows Cox proportional-hazards regression for survival. For patients who survive more than 6 months, the independent factors associated with poorer HCC survival are age >60 (hazard ratio[HR] = 1.19; 95% CI, 1.11–1.27), male (1.18; 1.10–1.27), AFP levels ≥400 (1.47; 1.37–1.58), positivity of HBsAg (1.10; 1.03–1.17), Child-Pugh B (1.32; 1.21–1.43) vs. A, HCC-alone (1.32; 1.09–1.59), prior (1.59; 1.23–2.05) and synchronous (1.52; 1.09–2.12) vs. metachronous group, respectively, treated with local ablation (2.08; 1.83–2.37), TAE (2.65; 2.39–2.93), radiotherapy (4.88; 4.09–5.81) and supportive care (3.70; 3.27–4.18) vs. surgery, respectively, TNM stage IIIA (1.20; 1.07–1.35) vs. I, and BCLC stages A (1.41; 1.23–1.63), B (1.98; 1.71–2.31), C (3.50; 2.80–4.39) and D (3.25; 2.61–4.04) vs. 0, respectively. The survival rates based on EHPM diagnostic year showed period 2003–2013(n = 337) was significant better than period 1986–2003(n = 233) (p < 0.001). Median survival time of period 2003–2013 and 1986–2003 was 3.83 and 1.92 years, respectively.

## Discussion

To our knowledge, the current study is the largest hospital-based study for EHPM over a 27-year period. EHPM incidence rate of the study is 3.91% (95% CI: 3.60–4.23%). Overall incidence rates of previous studies, which mostly enrolled all three subgroups of EHPM, were 2.1~25.7%^4^,^5^,^6^,^7^,^8^,^9^,^10^,^11^,^12^,^13^,^14^,^15^,^16^,^17^,^18^,^19^. Some limitations and potential bias exist in our study. Since the study was using medical records between 1986 and 2013 retrospectively, detection sensitivity of EHPM should be different among the study periods due to advance of imaging modalities. For diagnosis of HCC, diagnostic criteria was also different among study period 1986–2004 and 2005–2013 based on EASL^21^ and AASLD^22^ practice guidelines, respectively. Detection sensitivity should be improved due to advance of imaging modalities. The increasing incidence of EHPM over time might be possibly related to advance of modalities. Therefore, the important diagnostic methods were different among the study periods, diagnostic sensitivity and specificity was not consistent between 1986 and 2013.

Some cancer sites had relative higher percentage (>40%) among subgroups of EHPM (Table 3). Among three EHPM subgroups, prior group has the highest percentage (42.1%), the result is consistent with the majority of previous studies^8^,^9^,^13^,^14^,^15^,^17^,^19^. In prior group, higher percentage cancers sites that including cervical, breast, prostate and oropharyngeal cancers are low mortality to incidence ratio that represented lower fatality rates^27^. These cancers survivors had higher chances to suffer from HCC. In synchronous group, esophageal and biliary tract cancers had relative higher percentage. Kalaitzakis *et al.* reported cirrhotic patients, that common in our study population, had higher risk in these two cancers^28^. Among patients with esophageal cancer, 8 of 9 (89%) were heavy alcohol users which share common risk factors^29^. Among patients with biliary tract cancer, 4 of 7 cases (57%) were combined intrahepatic cholangiocarcinoma and HCC were diagnosed pathologically after tumor resection. Such rare combined cholangiocarcinoma and HCC possibly had been underestimated due to miss-diagnosis by image study in patients without tumor resection or liver transplantation^30^. In metachronous group, lung cancer presented with higher percentage with HCC, aging might play an important role.

Some differences in common cancer sites exist between eastern and western countries. For examples, the common EHPM in eastern countries were colon^3^,^6^,^7^,^8^,^11^,^16^,^17^, gastric^5^,^8^,^13^,^15^ and lung^3^,^16^ cancers. In contrast, the common EHPM in western countries were lung^10^, lymphoproliferative^9^, renal cell carcinoma^4^ and prostate^10^,^12^ cancers, as well as colon cancer^9^,^10^,^12^,^14^,^19^. Variation of geography and ethnic might be possible causes of the different distribution of EHPM, as well as higher-rank cancers. Some cancer sites of our study was similar with general population in Taiwan^31^, such as colon, lung, gastric and oropharyngeal cancers that are ranking as higher-order incident cancers. However, bladder and kidney cancers in EHPM, that ranked third and fourth in male, and ranked first and fifth in female, respectively, were not the top 10 cancer in Taiwan^31^. Although some previous studies reported that these two cancers were relative higher-order EHPM^4^,^8^,^12^,^15^,^19^, majority of these studies were at western countries that higher rank of these two cancers and relative small sample size to make a conclusion. Urogenital cancers of our study, kidney and bladder, were at unexpected higher ranks. The reasons for the higher rank of kidney and bladder cancers in our study are still unclear, the possible etiologies might be possible sharing same common factors (such as tobacco use, alcohol intake, and obesity), genetic predisposition, environmental factors, host effects, and interactions between these factors^32^. However, further investigation was needed to clarify this issue.

The current large scale hospital-based study has provided more convincing evidence in the clinical manifestations and survival rates of EHPM. Our study could give a more significant statistical power, and avoid the insignificant difference that related to the insufficient power. However, the study population still could not represent the geographic variation and ethnical difference of EHPM, because the patients were from the same medical center.

Mean age was older in EHPM group and the result was consistent with previous studies^8^,^10^,^20^, whereas some studies showed no significant difference^9^,^14^,^16^,^17^. Apparently, aging itself is an important risk factor of cancers incidence, the risk was definitely increased for those patients who were live longer. Higher rates of female patients in EHPM group might be related to lower rates in positivity of HBsAg and different distributions of cancer sites in these patients. Some studies showed no significant difference in gender^9^,^13^,^14^,^16^,^17^.

In comparison of liver functions, our EHPM patients have better liver functions reserve such as higher rates in Child-Pugh class A and higher albumin levels, and lower AST and ALT levels that reflected less hepatitis activity. Several previous reports showed that no significant difference in liver functions in cirrhosis^9^,^13^,^14^,^17^, ALT^13^,^14^ and prothrombin time^13^,^14^, whereas some other studies showed lower rates of cirrhosis^8^,^11^ and lower AST levels^14^ in EHPM group.

The prevalence rate of hepatitis B virus (HBV) and C (HCV) infections were lower in EHPM group. Majority of the prior studies have been demonstrated no difference in HBV^9^,^13^,^14^,^17^ and HCV^9^,^13^,^14^ infections. Onitsuka *et al.* showed that there was a lower rate of HBV^11^ infection in EHPM group. As we knew, HBV infection has been one of the poor prognostic factors for survival^33^. Higher AFP levels also implicated an important poor prognostic survival predictor^33^. Although there were no significant difference^9^,^13^,^14^ of AFP levels between both groups in some previous studies, our study showed lower rates of high AFP levels reflected less tumor invasive behavior of underlying HCC. Our EHPM patients have higher rates of earlier BCLC stage and higher chances to receive potential curative treatments such as surgery, so survival rates were higher. A previous study showed that no difference in BCLC staging between two groups but with a small case number were included^14^. The BCLC staging system offers prognostic prediction and treatment allocations for different HCC stages. The treatment schedules allocated for HCC patients demonstrated survival benefit in our previous study^34^.

Survival rates of EHPM improved especially after year 2003, these might be related to the improve survival of the both HCC and EHPM because of earlier cancers detection and improve cancers treatment over time. Furthermore, prognosis and survival of different cancers site might also influence EHPM survival. Therefore, the existent of EHPM should be an important issue for HCC survivors in the future.

Some studies showed no significant difference of survival between EHPM and HCC-alone groups^8^,^10^,^12^,^13^,^16^. Individual subgroups in the study showed that metachronous group benefit from better survival than all other subgroups after adjusting the bias and was also independent factor associated with higher survival rates. This might be explained that majority of patients in metachronous group were less severe HCC disease status.

There were different definitions and duration of diagnostic time of EHPM in previous studies. Majority of the studies defined prior, synchronous and metachronous groups with different duration that ranging from three months to one year. In addition, most of the studies did not separate the EHPM patients into subgroups due to relative small scale of cases. One study that compared the survival rate between prior combined metachronous and synchronous group^17^, the result showed the former survival was better. Survivals of these patients were mostly attributed by HCC related death^7^,^10^,^12^. As we knew, HCC was associated with significant high mortality to incidence ratio with a high fatality rate and poorer survival rates^27^. The presence of EHPM should be less impact in the mortality.

The factors associated with HCC survival were similar to our previous study. Young age, female, better liver functions reserve, less advanced TNM and BCLC stages, and received curative treatment with surgery were independent factors with better survival^33^,^34^,^35^.

Some limitations have been demonstrated in this study, some missing variables, co-morbidity disease, life style, occupational exposure and personal history such as tobacco use, alcohol consumption, or betel nut were not available in this retrospective study. Case-control study and design might be needed to further clarify the etiologies of EHPM.

In conclusion, EHPM patients were associated with less severe in both liver functions reserve and HCC tumor status. These patients have been benefit from better survival rates, these might be related to early diagnosis and improving treatment of HCC.

## Additional Information

**How to cite this article**: Kee, K. M. *et al.* Hepatocellular Carcinoma associated with Extra-hepatic Primary Malignancy: its Secular change, Clinical Manifestations and Survival. *Sci. Rep.* **6**, 30156; doi: 10.1038/srep30156 (2016).

## Figures and Tables

**Table 1:** Age-specific incidence rates (%) of extrahepatic primary malignancy, 1986–2013.

Age	N (1986–2013)	1986–89	1990–92	1993–95	1996–98	1999–2001	2002–04	2005–07	2008–10	2011–13
		n = 813	n = 865	n = 1277	n = 1747	n = 2341	n = 2104	n = 1900	n = 1665	n = 1560
<30	238	0.0	0.0	0.0	0.0	0.0	0.0	5.0	11.1	0.0
30–34	323	0.0	0.0	2.5	0.0	1.7	0.0	0.0	0.0	0.0
35–39	596	0.0	1.7	0.0	1.1	0.0	1.2	0.0	2.0	0.0
40–44	950	0.0	0.0	0.8	0.9	1.6	2.1	1.8	1.2	3.8
45–49	1305	0.8	1.0	0.9	2.8	1.2	2.0	3.5	5.0	4.8
50–54	1819	2.9	0.0	1.1	1.8	2.6	3.7	5.4	4.4	3.9
55–59	2179	0.7	1.5	0.0	2.9	1.5	3.8	5.1	5.6	6.0
60–64	2217	0.8	3.6	3.7	2.1	5.5	4.8	3.9	11.1	5.2
65–69	1996	3.3	4.9	6.4	4.5	4.6	4.0	4.5	6.6	6.4
70–74	1637	3.4	4.2	4.2	5.3	4.3	4.6	8.4	7.5	4.4
75–79	902	0.0	4.7	9.3	4.5	5.8	10.2	2.5	7.6	5.8
80–84	348		66.7	3.7	7.0	7.5	5.8	5.7	4.2	6.2
≥85	110	100.0		100.0	10.0	11.1	12.5	4.3	0.0	6.5
Age-standardized (30–84)		0.9	2.7	2.0	2.0	2.3	2.7	2.9	4.0	3.4
Crude rate (30–84)		1.5	2.3	2.6	3.0	3.3	4.1	4.6	6.2	5.1

**Table 2:** Age-specific average annual percentage change (AAPC) for 1986–2013.

Age (years)	Linear model		Model with quadratic trend term	
	AAPC for 1986–2013 (%)	p value	Sign of the second order term	p value
30–34	0.0	0.803	−	0.961
35–39	0.0	0.956	−	0.800
40–44	7.7	0.047	−	0.787
45–49	11.5	0.003	−	0.871
50–54	2.4	0.003	−	0.970
55–59	7.0	<0.001	−	0.905
60–64	5.0	0.001	−	0.404
65–69	6.0	0.338	−	0.913
70–74	2.4	0.170	−	0.897
75–79	12.1	0.620	−	0.214
80–84	—	0.300	+	0.001
Age-standardized	4.9	<0.001	−	0.624
Crude rate (30–84)	6.1	<0.001	−	0.735

**Table 3:** The common cancer site in extra-hepatic primary malignancy patients based on diagnostic time (n = 570).

No	Cancer sites of EHPM (top 10 cancer in Taiwan^31^)	Total number	Prior n = 240 (42.1%)	Synchronous n = 149 (26.1%)	Metachronous n = 181 (31.8%)
1	Colon (2)	81	35 (43.2)	19 (23.5)	27 (33.3)
2	Kidney	74	30 (40.5)	19 (25.7)	25 (33.8)
3	Bladder	67	30 (44.8)	14 (20.9)	23 (34.3)
4	Oropharynx (6)	63	34 (54)	16 (25.4)	13 (20.6)
5	Lung (4)	47	8 (17)	15 (31.9)	24 (51.1)
6	Gastric (7)	45	12 (26.7)	16 (35.6)	17 (37.8)
7	Prostate (5)	25	12 (48)	8 (32.0)	5 (20)
8	Breast (1)	24	16 (66.7)	4 (16.7)	4 (16.7)
9	Lymphoma	21	8 (38.1)	5 (23.8)	8 (38.1)
10	Cervix (8)	19	14 (73.7)	3 (15.8)	2 (10.5)
11	Nasopharynx	16	8 (50)	3 (18.8)	5 (31.3)
12	Esophagus	15	3 (20)	9 (60.0)	3 (20)
13	Biliary tract	12	0 (0)	7 (58.3)	5 (41.7)
14	Thyroid	11	8 (72.7)	1 (9.1)	2 (18.2)
15	Larynx	10	7 (70)	2 (20.0)	1 (10)
16	Skin (10)	9	5 (55.6)	3 (33.3)	1 (11.1)
17	Small intestine	7	1 (14.3)	2 (28.6)	4 (57.1)
18	Pancreas	6	2 (33.3)	0 (0)	4 (66.7)
19	Bone	5	1 (20)	1 (20)	3 (60)
20	Leukemia	4	2 (50)	0 (0)	2 (50)
21	Nasal sinus	3	0 (0)	1 (33.3)	2 (66.7)
22	Ovary	2	1 (50)	1 (50)	0 (0)
23	Multiple myeloma	2	1 (50)	0 (0)	1 (50)
24	Hodgkin’s disease	2	2 (100)	0 (0)	0 (0)

EHPM, extra-hepatic primary malignancy.

*Top 10 cancer in Taiwan^31^: rank 3, hepatocellular carcinoma; rank 9, uterus cancer.

**Table 4:** Univariate analysis shows clinical characteristics between extra-hepatic primary malignancy and hepatocellular carcinoma alone.

Variable (%)	EHPM (n = 570) (%)	Sex- and age-matched HCC-alone group (1:4) (n = 2280) (%)	HCC-alone group (n = 13985)(%*)	P1	P2
Age (years)	63.7 ± 10.6	63.7 ± 10.5	58.2 ± 12.7	0.990	<0.001
Male/Female	408 (71.6)/162 (28.4)	1632 (71.6)/648 (28.4)	10781 (77.1)/3204 (22.9)	1.000	0.002
AST (<40/≥40 IU/L)	194 (34.2)/374 (65.8)	557 (24.4)/1723 (75.6)	2622 (19.1)/11106 (80.9) (98.2%)	<0.001	<0.001
ALT (<40/≥40 IU/L)	272 (48.2)/292 (51.8)	854 (37.5)/1426 (62.5)	4680 (35.1)/8649 (64.9)(95.5%)	<0.001	<0.001
Bilirubin (<2/≥2 mg/dL)	507 (89.1)/62 (10.9)	1882 (82.5)/398 (17.5)	9574 (76.5)/2938 (23.5)(89.9%)	<0.001	<0.001
Albumin (<3.5/≥3.5 g/dl)	244 (42.9)/325 (57.1)	1091 (48.3)/1170 (51.7)	6139 (51.4)/5795 (48.6)(85.9%)	0.012	<0.001
AFP (<400/≥400 ng/mL)	427 (76.8)/129 (23.2)	1519 (66.6)/761 (33.4)	7897 (58.2)/5683 (41.8)(97.1%)	<0.001	<0.001
Child-Pugh (A/B/C)	432 (75.8)/122 (21.4)/16 (2.8)	1486 (65.2)/644 (28.2)/150 (66)	6277 (59.5)/3131 (29.7)/1136 (10.8) (76.4%)	<0.001	<0.001
HBsAg (−/+)	326 (58.4)/232 (41.6)	1222 (53.6)/1058 (46.4)	5531 (41.7)/7719 (58.3)(94.9%)	0.040	<0.001
Anti-HCV Ab (−/+)	328 (58.8)/230 (41.2)	1257 (55.1)/1023 (44.9)	7038 (60.4)/4608 (39.6) (83.8%)	0.120	0.436
7th TNM staging			(87.5%)	<0.001	<0.001
I	300 (52.6)	978 (42.9)	4890 (40.2)		
II	123 (21.6)	508 (22.3)	2503 (20.6)		
IIIA	60 (10.5)	195 (8.6)	1347 (11.1)		
IIIB	47 (8.2)	306 (13.4)	2200 (18.1)		
IIIC	18 (3.2)	132 (5.8)	587 (4.8)		
IVA	1 (0.2)	1 (0.04)	8 (0.1)		
IVB	21 (3.7)	160 (7.0)	628 (5.2)		
BCLC staging			(76.0%)	<0.001	<0.001
0	51 (8.9)	202 (8.9)	708 (6.7)		
A	243 (42.6)	712 (31.2)	2662 (25.4)		
B	165 (28.9)	642 (28.2)	3096 (29.5)		
C	95 (16.7)	574 (25)	2887 (27.5)		
D	16 (2.8)	2150 (6.5)	1136 (10.8)		
Initial treatement of HCC				0.083	<0.001
Transplantation	0 (0)	2 (0.1)	8 (0.1)		
Surgery	114 (20.0)	359 (15.7)	1711 (12.2)		
Local ablation**	106 (18.6)	384 (16.8)	1285 (9.2)		
TAE	208 (36.5)	870 (38.2)	4082 (29.2)		
R/T or C/T	28 (4.9)	160 (7.0)	833 (6.0)		
Supportive	114 (20.0)	505 (22.1)	6066 (43.4)		

Abbr: EHPM, extra-hepatic primary malignancy; HCC, hepatocellular carcinoma; AFP, alpha fetoprotein; TAE, Transcatheter arterial embolization; R/T, radiotherapy; C/T, chemotherapy, BCLC, Barcelona Clinic Liver Cancer. P1, p value (EHPM vs. matched HCC-alone); P2, p value (EHPM vs. all HCC-alone).

^*^Available data for analysis.

^**^Local ablation include radiofrequency ablation, percutaneous ethanol injection, percutaneous acetic acid injection, percutaneous microwave coagulation therapy.

**Table 5:** Multivariate analysis demonstrated the independent factors associated with extra-hepatic primary malignancy.

Variable	All patients (n = 5180)			EHPM and four times sex- and age-matched HCC-alone cases (n = 1485)	
	Comparisons	Odds ratio 1 (95% C.I.)	P value	Odds ratio 2 (95% C.I.)	P value
Age (years)	>60 vs. ≤60	1.74 (1.43–2.10)	<0.001	—	—
AST levels (IU/L)	<40 vs. ≥40	1.32 (1.08–1.61)	0.007	—	—
ALT levels (IU/L)	<40 vs. ≥40	—	—	1.43 (1.18–1.74)	<0.001
Child-Pugh class	A vs. B	1.43 (1.14–1.80)	0.002	1.46 (1.15–1.87)	0.002
BCLC staging	0 vs. D	2.56 (1.32–4.99)	0.006	1.64 (0.82–3.29)	0.162
	A vs. D	3.42 (1.87–6.24)	<0.001	2.33 (1.25–4.35)	0.008
	B vs. D	2.27 (1.24–4.15)	0.008	1.79 (0.96–3.35)	0.069
	C vs. D	1.73 (0.94–3.16)	0.077	1.34 (0.72–2.51)	0.361
AFP (ng/ml)	<400 vs. ≥400	1.38 (1.11–1.72)	0.003	—	—
Anti-HCV	− vs. +	1.49 (1.20–1.87)	<0.001	—	—
HBsAg	− vs. +	1.70 (1.36–2.11)	<0.001	—	—

Abbr.: EHPM, extra-hepatic primary malignancy; BCLC, Barcelona clinic liver cancer.

**Table 6:** Multivariate analysis for factors associated with survival of hepatocellular carcinoma by Cox-proportional hazards regression.

Variable	Comparisons	Hazard ratio 1 (95% C.I.)	P1 value	Hazard ratio 2 (95% C.I.)	P2 value
Each individual EHPM and HCC-alone groups	HCC-alone vs. Metachronous	1.54 (1.28–1.86)	<0.001	1.32 (1.09–1.59)	0.004
	Prior vs. Metachronous	1.76 (1.38–2.24)	<0.001	1.59 (1.23–2.05)	<0.001
	Synchronous vs. Metachronous	1.62 (1.22–2.14)	0.001	1.52 (1.09–2.12)	0.013
Age (years)	>60 vs. ≤60	1.12 (1.06–1.18)	<0.001	1.19 (1.11–1.27)	<0.001
Gender	Male vs. Female	1.15 (1.09–1.22)	<0.001	1.18 (1.10–1.27)	<0.001
AST levels (IU/L)	≥40 vs. <40	1.43 (1.32–1.54)	<0.001	1.45 (1.32–1.59)	<0.001
ALT levels (IU/L)	<40 vs. ≥40	1.08 (1.02–1.14)	0.010	1.19 (1.10–1.28)	<0.001
Total bilirubin (mg/dL)	≥2 vs. <2	1.11 (1.03–1.19)	0.005		
Albumin (g/dL)	<3.5 vs. ≥3.5	1.23 (1.15–1.30)	<0.001	1.29 (1.19–1.39)	<0.001
AFP (ng/mL)	≥400 vs. <400	1.64 (1.56–1.73)	<0.001	1.47 (1.37–1.58)	<0.001
HBsAg	+vs. −	1.17 (1.11–1.23)	<0.001	1.10 (1.03–1.17)	0.006
7th TNM staging	II vs. I	0.95 (0.89–1.02)	0.187	0.96 (0.88–1.04)	0.293
	IIIA vs. I	1.28 (1.17–1.40)	<0.001	1.20 (1.07–1.35)	0.003
	IIIB vs. I	1.79 (1.59–2.01)	<0.001	0.82 (0.67–1.00)	0.053
	IIIC vs. I	1.71 (1.48–1.97)	<0.001	0.85 (0.67–1.08)	0.174
	IV vs. I	2.22 (1.94–2.55)	<0.001	1.10 (0.86–1.42)	0.454
BCLC staging	A vs. 0	1.34 (1.17–1.54)	<0.001	1.41 (1.23–1.63)	<0.001
	B vs. 0	2.01 (1.74–2.32)	<0.001	1.98 (1.71–2.31)	<0.001
	C vs. 0	2.38 (2.00–2.83)	<0.001	3.50 (2.80–4.39)	<0.001
	D vs. 0	5.29 (4.44–6.30)	<0.001	3.25 (2.61–4.04)	<0.001
Child-Pugh class	B vs. A	1.51 (1.41–1.62)	<0.001	1.32 (1.21–1.43)	<0.001
Initial treatement of HCC	Local ablation vs. Surgery	2.07 (1.83–2.33)	<0.001	2.08 (1.83–2.37)	<0.001
	TAE vs. Surgery	2.57 (2.34–2.82)	<0.001	2.65 (2.39–2.93)	<0.001
	R/T or C/T vs. Surgery	3.43 (3.02–3.88)	<0.001	4.88 (4.09–5.81)	<0.001
	Supportive vs. Surgery	5.42 (4.90–5.99)	<0.001	3.70 (3.27–4.18)	<0.001

Hazards ratio 1 and p1 value, all patients; Hazard ratio 2 and p2 value included patients who survive more than 6 months.

Abbr: EHPM, extra-hepatic primary malignancy; HCC, hepatocellular carcinoma; AFP, alpha fetoprotein; TAE, Transcatheter arterial embolization; R/T, radiotherapy; C/T, chemotherapy, BCLC, Barcelona Clinic Liver Cancer.
